# Supplementary material for: Distinct roles for CKM–Mediator in controlling Polycomb-dependent chromosomal interactions and priming genes for induction
Source: Nat Struct Mol Biol. 2022 Oct 11;29(10):1000–10. doi: 10.1038/s41594-022-00840-5 (PMC9568430; doi:10.1038/s41594-022-00840-5)
Supplement: Supplementary file 1 — Reporting Summary [file 41594_2022_840_MOESM1_ESM.pdf]

nature portfolio

Corresponding author(s): Emilia Dimitrova, Robert J. Klose

Last updated by author(s): 24/05/22

Reporting Summary

Nature Portfolio wishes to improve the reproducibility of the work that we publish. This form provides structure for consistency and transparency in reporting. For further information on Nature Portfolio policies, see our Editorial Policies and the Editorial Policy Checklist.

Statistics

For all statistical analyses, confirm that the following items are present in the figure legend, table legend, main text, or Methods section.

n/a	Confirmed	
☐	☒	The exact sample size (*n*) for each experimental group/condition, given as a discrete number and unit of measurement
☐	☒	A statement on whether measurements were taken from distinct samples or whether the same sample was measured repeatedly
☐	☒	The statistical test(s) used AND whether they are one- or two-sided<br>*Only common tests should be described solely by name; describe more complex techniques in the Methods section.*
☒	☐	A description of all covariates tested
☒	☐	A description of any assumptions or corrections, such as tests of normality and adjustment for multiple comparisons
☐	☒	A full description of the statistical parameters including central tendency (e.g. means) or other basic estimates (e.g. regression coefficient) AND variation (e.g. standard deviation) or associated estimates of uncertainty (e.g. confidence intervals)
☐	☒	For null hypothesis testing, the test statistic (e.g. *F*, *t*, *r*) with confidence intervals, effect sizes, degrees of freedom and *P* value noted<br>*Give P values as exact values whenever suitable.*
☒	☐	For Bayesian analysis, information on the choice of priors and Markov chain Monte Carlo settings
☒	☐	For hierarchical and complex designs, identification of the appropriate level for tests and full reporting of outcomes
☐	☒	Estimates of effect sizes (e.g. Cohen's *d*, Pearson's *r*), indicating how they were calculated

Our web collection on statistics for biologists contains articles on many of the points above.

Software and code

Policy information about availability of computer code

Data collection	Image Studio v5.2 (LI-COR)
Data analysis	R version 4.0.2 (R Core Team, 2020), Bowtie 2 (Langmead and Salzberg, 2012), STAR v2.5.4 (Dobin et al., 2013), MACS2 v2.1.1 (Zhang et al., 2008), Sambamba (Tarasov et al., 2015), SAMtools v1.7 (Li et al., 2009), deepTools v3.1.1 (Ramirez et al., 2016), BEDtools v2.17.0 (Quinlan and Hall, 2010), DESeq2 (Love et al., 2014), Hi-C Pro 2.9 (Servant et al., 2015), GENOVA (van der Weide et al., 2021, github.com/deWitLab/GENOVA), HiCUP (Wingett et al., 2015), CHiCAGO (Cairns et al., 2016), CapSequm2 (Hughes et al., 2014), https://github.com/nFursova/Calibrated_ChIPseq_RNAseq

For manuscripts utilizing custom algorithms or software that are central to the research but not yet described in published literature, software must be made available to editors and reviewers. We strongly encourage code deposition in a community repository (e.g. GitHub). See the Nature Portfolio guidelines for submitting code & software for further information.

Data

Policy information about availability of data

All manuscripts must include a data availability statement. This statement should provide the following information, where applicable:

- Accession codes, unique identifiers, or web links for publicly available datasets
- A description of any restrictions on data availability
- For clinical datasets or third party data, please ensure that the statement adheres to our policy

The datasets generated in this study are available from GEO database under accession number GSE185930. Published data used in this study include mouse ESC TAD and loop coordinates (GSE96107), Polycomb domains (GSE119620), CDK8 peaks (GSE98756), and H3K27Ac peaks (GSE136424). For cnRNA-seq processing we

used mm10 (GenBank: BK000964.3, https://www.ncbi.nlm.nih.gov/nuccore/bk000964) and dm6 (GenBank: M21017.1, https://www.ncbi.nlm.nih.gov/nuccore/M21017.1) rDNA genomic datasets.

Field-specific reporting

Please select the one below that is the best fit for your research. If you are not sure, read the appropriate sections before making your selection.

☒ Life sciences ☐ Behavioural & social sciences ☐ Ecological, evolutionary & environmental sciences

For a reference copy of the document with all sections, see nature.com/documents/nr-reporting-summary-flat.pdf

Life sciences study design

All studies must disclose on these points even when the disclosure is negative.

Sample size	Sample sizes were determined based on previous studies performed in the lab using similar techniques to enable reasonable statistical analysis.
Data exclusions	No data were excluded
Replication	Reported experimental findings were reproducible in multiple independent biological replicates. c-ChIPseq and CapC experiments were performed in triplicates, cnRNA-seq - in quadruplicates, and Hi-C in duplicates. The numbers of biological replicates for each experiment are given in Methods section and/or in the figure legends.
Randomization	Randomization was not relevant for this study as it includes only molecular assays performed in cell lines of known genotype.
Blinding	Blinding was not relevant for this study as there were no prior assumptions about experimental outcomes. All data was collected and processed uniformly regardless of treatment groups.

Reporting for specific materials, systems and methods

We require information from authors about some types of materials, experimental systems and methods used in many studies. Here, indicate whether each material, system or method listed is relevant to your study. If you are not sure if a list item applies to your research, read the appropriate section before selecting a response.

Materials & experimental systems

n/a	Involved in the study	
☐	☒	Antibodies
☐	☒	Eukaryotic cell lines
☒	☐	Palaeontology and archaeology
☒	☐	Animals and other organisms
☒	☐	Human research participants
☒	☐	Clinical data
☒	☐	Dual use research of concern

Methods

n/a	Involved in the study	
☐	☒	ChIP-seq
☒	☐	Flow cytometry
☒	☐	MRI-based neuroimaging

Antibodies

Antibodies used	All anibodies used are listed in Supplementary Table
Validation	anti-CDK8 (ChIP-seq) - validated in Pelish et al 2015<br>anti-H3K27me3 - validated in Rose at al. 2016<br>anti-RING1B - manufacturer-validated against various cell lines by western blot, validated in the Klose lab in a conditional knock-out line by ChIP-seq and western blot (Fursova et al 2019)<br>anti-SUZ12 - manufacturer-validated against various cell lines by western blot, validated in the Klose lab in a conditional knock-out cell line (Dobrinic et al 2020)<br>anti-PCGF2 - manufacturer-validated, validated in the Klose lab in a conditional knock-out line by ChIP-seq and western blot (Fursova et al. 2019)<br>anti-CBX7 (ChIPseq) - manufacturer-validated, validated in the Klose lab in a conditional knock-out line by ChIP-seq and western blot (Fursova et al. 2019)<br>anti-CBX7 (Western blot) - manufacturer-validated by western blot, 7 citations: https://www.citeab.com/antibodies/221872-07-981-anti-cbx7-antibody?des=c3ab80f14feb824d<br>anti-T7-tag - validated here western blot with extracts from an untagged cell line by ChIPseq in a degron cell line (unpublished). Validated for ChIPseq in Brown et al 2017.<br>anti-TBP - manufacturer validated in various cell types by cellular fractionation, 235 citations: https://www.citeab.com/antibodies/753557-ab818-anti-tata-binding-protein-tbp-antibody-1tbp18?des=1ee5e4f398055d5b<br>anti-MED12 (54 citations https://www.citeab.com/antibodies/655615-a300-774a-rabbit-anti-med12-antibody-affinity-purifi?

des=0e1bb45fbb1bfc17), anti-MED1 (92 citations https://www.citeab.com/antibodies/655647-a300-793a-rabbit-anti-med1-antibody-affinity-purifie?des=d802665adf8028ae), anti-MED14 (7 citations https://www.citeab.com/antibodies/654594-a301-044a-rabbit-anti-crsp2-drip150-antibody-affinit), anti-MED15 (6 citations https://www.citeab.com/antibodies/656694-a302-422a-rabbit-anti-med15-antibody-affinity-purifi?des=1f0dd4dd056c9dc6), anti-MED23 (13 citations https://www.citeab.com/antibodies/655005-a300-425a-rabbit-anti-crsp3-antibody-affinity-purifi?des=3dab3084bccb71ae) - manufacturer validated by IP and western blot
anti-MED17 - manufacturer validated by IF and Western blot using whole cell extract.
anti-MED13 - manufacturer validated by western blot and IP, validated here in a conditional knock-out cell line
anti-MED13L - manufacturer validated by western blot and IP, validated here in a conditional knock-out cell line
anti-CDK8 (Western blot) - validated by Western blot in the Klose lab in a knock-out cell line (unpublished)
anti-HDAC1 - manufacturer-validated by Western blot; 32 citations https://www.citeab.com/antibodies/762876-ab109411-anti-hdac1-antibody-epr460-2?des=ca96abb53494f759

Eukaryotic cell lines

Policy information about cell lines

Cell line source(s)	All mES cell lines used in this study were generated in the Klose lab:<br>CDK-MED cKO (Med13/13l fl/fl) mouse embryonic stem cell line (Dimitrova et al., 2018)<br>cPRC1 cKO (Pcgf4-/- Pcgf2 fl/fl) mouse embryonic stem cell line (Fursova et al., 2019)<br>TetR-PCGF2 TOT2N mouse embryonic stem cell line (with TetO integration) (Blackledge et al., 2014)<br>TetR-GFP TOT2N mouse embryonic stem cell line (with TetO integration) (Blackledge et al., 2014)<br>TetR-CDK8 TOT2N mouse embryonic stem cell line (with TetO integration) - this study<br>Med13/13l fl/fl Med14-T7 - mouse embryonic stem cell line generated in this study<br>Human HEK293T or drosophila SG4 cells (sourced from ATCC) were used as material for calibration but not as an experimental system.
Authentication	All cell lines generated in this study were validated by PCR, sequencing and Western blot. All cell lines generated for previous studies (Dimitrova et al, 2018; Blackledge et al, 2014; Fursova et al, 2019) were validated in their respective publication and confirmed in this study by Western blot.
Mycoplasma contamination	All cell lines were regularly tested for mycoplasma contamination and confirmed to be negative.
Commonly misidentified lines<br>(See ICLAC register)	No commonly misidentified cell lines were used.

ChIP-seq

Data deposition

☒ Confirm that both raw and final processed data have been deposited in a public database such as GEO.

☒ Confirm that you have deposited or provided access to graph files (e.g. BED files) for the called peaks.

Data access links<br>*May remain private before publication.*	https://www.ncbi.nlm.nih.gov/geo/query/acc.cgi?acc=GSE185930
Files in database submission	01-Med13fl-ESC-MED14-T7-UNT-rep1_R1.fastq.gz<br>01-Med13fl-ESC-MED14-T7-UNT-rep1_R2.fastq.gz<br>02-Med13fl-ESC-MED14-T7-TAM-rep1_R1.fastq.gz<br>02-Med13fl-ESC-MED14-T7-TAM-rep1_R2.fastq.gz<br>03-Med13fl-ESC-MED14-T7-UNT-rep2_R1.fastq.gz<br>03-Med13fl-ESC-MED14-T7-UNT-rep2_R2.fastq.gz<br>04-Med13fl-ESC-MED14-T7-TAM-rep2_R1.fastq.gz<br>04-Med13fl-ESC-MED14-T7-TAM-rep2_R2.fastq.gz<br>05-Med13fl-ESC-MED14-T7-UNT-rep3_R1.fastq.gz<br>05-Med13fl-ESC-MED14-T7-UNT-rep3_R2.fastq.gz<br>06-Med13fl-ESC-MED14-T7-TAM-rep3_R1.fastq.gz<br>06-Med13fl-ESC-MED14-T7-TAM-rep3_R2.fastq.gz<br>07-Med13fl-RA-MED14-T7-UNT-rep1_R1.fastq.gz<br>07-Med13fl-RA-MED14-T7-UNT-rep1_R2.fastq.gz<br>08-Med13fl-RA-MED14-T7-TAM-rep1_R1.fastq.gz<br>08-Med13fl-RA-MED14-T7-TAM-rep1_R2.fastq.gz<br>09-Med13fl-RA-MED14-T7-UNT-rep2_R1.fastq.gz<br>09-Med13fl-RA-MED14-T7-UNT-rep2_R2.fastq.gz<br>10-Med13fl-RA-MED14-T7-TAM-rep2_R1.fastq.gz<br>10-Med13fl-RA-MED14-T7-TAM-rep2_R2.fastq.gz<br>10-PCGF2-TAM-B1_R1.fastq.gz<br>10-PCGF2-TAM-B1_R2.fastq.gz<br>11-Med13fl-RA-MED14-T7-UNT-rep3_R1.fastq.gz<br>11-Med13fl-RA-MED14-T7-UNT-rep3_R2.fastq.gz<br>11-PCGF2-TAM-B2_R1.fastq.gz<br>11-PCGF2-TAM-B2_R2.fastq.gz<br>12-Med13fl-RA-MED14-T7-TAM-rep3_R1.fastq.gz<br>12-Med13fl-RA-MED14-T7-TAM-rep3_R2.fastq.gz

12-PCGF2-TAM-B3_R1.fastq.gz
12-PCGF2-TAM-B3_R2.fastq.gz
13-Med13fl-ESC-Inp-UNT-rep1_R1.fastq.gz
13-Med13fl-ESC-Inp-UNT-rep1_R2.fastq.gz
14-Med13fl-ESC-Inp-TAM-rep1_R1.fastq.gz
14-Med13fl-ESC-Inp-TAM-rep1_R2.fastq.gz
15-Med13fl-ESC-Inp-UNT-rep2_R1.fastq.gz
15-Med13fl-ESC-Inp-UNT-rep2_R2.fastq.gz
16-Med13fl-ESC-Inp-TAM-rep2_R1.fastq.gz
16-Med13fl-ESC-Inp-TAM-rep2_R2.fastq.gz
17-Med13fl-ESC-Inp-UNT-rep3_R1.fastq.gz
17-Med13fl-ESC-Inp-UNT-rep3_R2.fastq.gz
18-Med13fl-ESC-Inp-TAM-rep3_R1.fastq.gz
18-Med13fl-ESC-Inp-TAM-rep3_R2.fastq.gz
19-Med13fl-RA-Inp-UNT-rep1_R1.fastq.gz
19-Med13fl-RA-Inp-UNT-rep1_R2.fastq.gz
1-RING1b-UNT-B1_R1.fastq.gz
1-RING1b-UNT-B1_R2.fastq.gz
20-Med13fl-RA-Inp-TAM-rep1_R1.fastq.gz
20-Med13fl-RA-Inp-TAM-rep1_R2.fastq.gz
21-Med13fl-RA-Inp-UNT-rep2_R1.fastq.gz
21-Med13fl-RA-Inp-UNT-rep2_R2.fastq.gz
22-Med13fl-RA-Inp-TAM-rep2_R1.fastq.gz
22-Med13fl-RA-Inp-TAM-rep2_R2.fastq.gz
23-Med13fl-RA-Inp-UNT-rep3_R1.fastq.gz
23-Med13fl-RA-Inp-UNT-rep3_R2.fastq.gz
24-Med13fl-RA-Inp-TAM-rep3_R1.fastq.gz
24-Med13fl-RA-Inp-TAM-rep3_R2.fastq.gz
25-INP-UNT-B1_R1.fastq.gz
25-INP-UNT-B1_R2.fastq.gz
26-INP-UNT-B2_R1.fastq.gz
26-INP-UNT-B2_R2.fastq.gz
27-INP-UNT-B3_R1.fastq.gz
27-INP-UNT-B3_R2.fastq.gz
28-INP-TAM-B1_R1.fastq.gz
28-INP-TAM-B1_R2.fastq.gz
29-INP-TAM-B2_R1.fastq.gz
29-INP-TAM-B2_R2.fastq.gz
2-RING1B-UNT-B2_R1.fastq.gz
2-RING1B-UNT-B2_R2.fastq.gz
31-INP-TAM-B3_R1.fastq.gz
31-INP-TAM-B3_R2.fastq.gz
35-Med13fl-RA-UNT-Cdk8-rep1_R1.fastq.gz
35-Med13fl-RA-UNT-Cdk8-rep1_R2.fastq.gz
36-Med13fl-RA-UNT-Cdk8-rep2_R1.fastq.gz
36-Med13fl-RA-UNT-Cdk8-rep2_R2.fastq.gz
3-RING1B-UNT-B3_R1.fastq.gz
3-RING1B-UNT-B3_R2.fastq.gz
42-Med13fl-RA-UNT-Cdk8-rep3_R1.fastq.gz
42-Med13fl-RA-UNT-Cdk8-rep3_R2.fastq.gz
4-RING1B-TAM-B1_R1.fastq.gz
4-RING1B-TAM-B1_R2.fastq.gz
5-RING1B-TAM-B2_R1.fastq.gz
5-RING1B-TAM-B2_R2.fastq.gz
6-RING1B-TAM-B3_R1.fastq.gz
6-RING1B-TAM-B3_R2.fastq.gz
7-PCGF2-UNT-B1_R1.fastq.gz
7-PCGF2-UNT-B1_R2.fastq.gz
8-PCGF2-UNT-B2_R1.fastq.gz
8-PCGF2-UNT-B2_R2.fastq.gz
9-PCGF2-UNT-B3_R1.fastq.gz
9-PCGF2-UNT-B3_R2.fastq.gz
lib18-Med13fl-UNT-Rep1-CDK8-ChIPseq_R1.fastq.gz
lib18-Med13fl-UNT-Rep1-CDK8-ChIPseq_R2.fastq.gz
lib20-Med13fl-TAM-Rep1-CDK8-ChIPseq_R1.fastq.gz
lib20-Med13fl-TAM-Rep1-CDK8-ChIPseq_R2.fastq.gz
lib21-Med13fl-UNT-Rep3-CDK8-ChIPseq_R1.fastq.gz
lib21-Med13fl-UNT-Rep3-CDK8-ChIPseq_R2.fastq.gz
lib22-Med13fl-TAM-Rep3-CDK8-ChIPseq_R1.fastq.gz
lib22-Med13fl-TAM-Rep3-CDK8-ChIPseq_R2.fastq.gz
lib23-Med13fl-UNT-RepB1-CDK8-ChIPseq_R1.fastq.gz
lib23-Med13fl-UNT-RepB1-CDK8-ChIPseq_R2.fastq.gz
lib24-Med13fl-TAM-RepB1-CDK8-ChIPseq_R1.fastq.gz
lib24-Med13fl-TAM-RepB1-CDK8-ChIPseq_R2.fastq.gz
MED13fl-K27me3-TAM-B1_R1.fastq.gz
MED13fl-K27me3-TAM-B1_R2.fastq.gz

MED13fl-K27me3-TAM-B2_R1.fastq.gz
MED13fl-K27me3-TAM-B2_R2.fastq.gz
MED13fl-K27me3-TAM-B3_R1.fastq.gz
MED13fl-K27me3-TAM-B3_R2.fastq.gz
MED13fl-K27me3-UNT-B1_R1.fastq.gz
MED13fl-K27me3-UNT-B1_R2.fastq.gz
MED13fl-K27me3-UNT-B2_R1.fastq.gz
MED13fl-K27me3-UNT-B2_R2.fastq.gz
MED13fl-K27me3-UNT-B3_R1.fastq.gz
MED13fl-K27me3-UNT-B3_R2.fastq.gz
MED13fl-NativeINP-TAM-B1_R1.fastq.gz
MED13fl-NativeINP-TAM-B1_R2.fastq.gz
MED13fl-NativeINP-TAM-B2_R1.fastq.gz
MED13fl-NativeINP-TAM-B2_R2.fastq.gz
MED13fl-NativeINP-TAM-B3_R1.fastq.gz
MED13fl-NativeINP-TAM-B3_R2.fastq.gz
MED13fl-NativeInp-UNT-B1_R1.fastq.gz
MED13fl-NativeInp-UNT-B1_R2.fastq.gz
MED13fl-NativeInp-UNT-B2_R1.fastq.gz
MED13fl-NativeInp-UNT-B2_R2.fastq.gz
MED13fl-NativeInp-UNT-B3_R1.fastq.gz
MED13fl-NativeInp-UNT-B3_R2.fastq.gz
PCGF2fl-TAM-Rep1-CDK8-ChIPseq_R1.fastq.gz
PCGF2fl-TAM-Rep1-CDK8-ChIPseq_R2.fastq.gz
PCGF2fl-TAM-Rep1-XInput_R1.fastq.gz
PCGF2fl-TAM-Rep1-XInput_R2.fastq.gz
PCGF2fl-TAM-Rep2-CDK8-ChIPseq_R1.fastq.gz
PCGF2fl-TAM-Rep2-CDK8-ChIPseq_R2.fastq.gz
PCGF2fl-TAM-Rep2-XInput_R1.fastq.gz
PCGF2fl-TAM-Rep2-XInput_R2.fastq.gz
PCGF2fl-TAM-Rep3-CDK8-ChIPseq_R1.fastq.gz
PCGF2fl-TAM-Rep3-CDK8-ChIPseq_R2.fastq.gz
PCGF2fl-TAM-Rep3-XInput_R1.fastq.gz
PCGF2fl-TAM-Rep3-XInput_R2.fastq.gz
PCGF2fl-UNT-Rep1-CDK8-ChIPseq_R1.fastq.gz
PCGF2fl-UNT-Rep1-CDK8-ChIPseq_R2.fastq.gz
PCGF2fl-UNT-Rep1-XInput_R1.fastq.gz
PCGF2fl-UNT-Rep1-XInput_R2.fastq.gz
PCGF2fl-UNT-Rep2-CDK8-ChIPseq_R1.fastq.gz
PCGF2fl-UNT-Rep2-CDK8-ChIPseq_R2.fastq.gz
PCGF2fl-UNT-Rep2-XInput_R1.fastq.gz
PCGF2fl-UNT-Rep2-XInput_R2.fastq.gz
PCGF2fl-UNT-Rep3-CDK8-ChIPseq_R1.fastq.gz
PCGF2fl-UNT-Rep3-CDK8-ChIPseq_R2.fastq.gz
PCGF2fl-UNT-Rep3-XInput_R1.fastq.gz
PCGF2fl-UNT-Rep3-XInput_R2.fastq.gz
MED1313Lfl_K27me3_TAM_mm10_spikeinnormalised_MERGED.MACS2.bw
MED1313Lfl_K27me3_UNT_mm10_spikeinnormalised_MERGED.MACS2.bw
MED1313Lfl_PCGF2_TAM_mm10_spikeinnormalised_MERGED_MACS2.bw
MED1313Lfl_PCGF2_UNT_mm10_spikeinnormalised_MERGED_MACS2.bw
MED1313Lfl_RING1B_TAM_mm10_spikeinnormalised_MERGED_MACS2.bw
MED1313Lfl_RING1B_UNT_mm10_spikeinnormalised_MERGED_MACS2.bw
Med13fl_ESC_TAM_MED14-T7_mm10_readcountnorm_MERGED_MACS2.bw.bw
Med13fl_ESC_UNT_MED14-T7_mm10_readcountnorm_MERGED_MACS2.bw.bw
Med13fl_RA_TAM_MED14-T7_mm10_readcountnorm_MERGED_MACS2.bw.bw
Med13fl_RA_UNT_MED14-T7_mm10_readcountnorm_MERGED_MACS2.bw.bw
mESC_MED13fl_TAM_CDK8_mm10.UniqMapped_downsampled_MERGED_MACS2.bw.bw
mESC_MED13fl_UNT_CDK8_mm10.UniqMapped_downsampled_MERGED_MACS2.bw.bw
mESC_Pcgf2fl_XChIP_TAM_CDK8_mm10.UniqMapped_downsampled_MERGED_MACS2.bw.bw
mESC_Pcgf2fl_XChIP_UNT_CDK8_mm10.UniqMapped_downsampled_MERGED_MACS2.bw.bw
Med13fl_RA_UNT_CDK8_mm10_spikeinnormalised.MERGED_downsampled.MACS2.bw
MED1313Lfl_K27me3_UNT_mm10_spikeinnormalised_MERGED.MACS2.bw
MED1313Lfl_K27me3_TAM_mm10_spikeinnormalised_MERGED.MACS2.bw
22_Med13fl_ESC_UNT_CBX7_ChIPseq_rep1_R1.fastq.gz
22_Med13fl_ESC_UNT_CBX7_ChIPseq_rep1_R2.fastq.gz
23_Med13fl_ESC_TAM_CBX7_ChIPseq_rep1_R1.fastq.gz
23_Med13fl_ESC_TAM_CBX7_ChIPseq_rep1_R2.fastq.gz
24_Med13fl_ESC_UNT_CBX7_ChIPseq_rep2_R1.fastq.gz
24_Med13fl_ESC_UNT_CBX7_ChIPseq_rep2_R2.fastq.gz
26_Med13fl_ESC_TAM_CBX7_ChIPseq_rep2_R1.fastq.gz
26_Med13fl_ESC_TAM_CBX7_ChIPseq_rep2_R2.fastq.gz
27_Med13fl_ESC_UNT_CBX7_ChIPseq_rep3_R1.fastq.gz
27_Med13fl_ESC_UNT_CBX7_ChIPseq_rep3_R2.fastq.gz
28_Med13fl_ESC_TAM_CBX7_ChIPseq_rep3_R1.fastq.gz
28_Med13fl_ESC_TAM_CBX7_ChIPseq_rep3_R2.fastq.gz
MED13fl_CBX7_UNT_mm10_spikeinnormalised_MERGED.MACS2.bw

MED13fl_CBX7_TAM_mm10_spikeinnormalised_MERGED.MACS2.bw

Genome browser session (e.g. UCSC)	All merged bigWig files were uploaded to GEO: https://www.ncbi.nlm.nih.gov/geo/query/acc.cgi?acc=GSE185930

Methodology

Replicates	All ChIP-seq experiments were performed in biological triplicates
Sequencing depth	All libraries were sequenced as 40bp paired-end reads. Number of reads is given in Supplementary Table.
Antibodies	Rabbit polyclonal anti-CDK8 Bethyl laboratories Cat# A302-500A lot 2<br>Rabbit monoclonal anti-RING1B Cell Signaling Technology Cat# 5694 lot 3<br>Rabbit polyclonal anti-PCGF2 (Mel-18 H-115) Santa Cruz Cat# sc-10744 lot D0903<br>Rabbit polyclonal anti-H3K27me3 In house (Rose et al., 2016)<br>Rabbit monoclonal anti-T7-Tag (D9E1X) Cell Signaling Technology Cat# 13246 lot 1<br>Rabbit monoclonal anti-CBX7, abcam Cat#ab21873 lot GR3210651-1
Peak calling parameters	all peaks used in this study were published previously (Fursova et al, 2019; Dimitrova et al, 2018; Feldmann et al, 2020)
Data quality	Quality of ChIP-seq data was assessed by visual inspection of individual replicate bigWig files and comparison with other published data sets, as well as by metaplot, heatmap and correlation analysis using deepTools.
Software	Paired-end reads were aligned to the concatenated mouse (mm10) and spike-in (dm6 for native, hg19 for cross-linked cChIP-seq) genome sequences using Bowtie 2 ("–no-mixed" and "–no-discordant" options). Only uniquely mapped reads were kept for downstream analysis, after removal of PCR duplicates with Sambamba. Genome coverage tracks were generated using the pileup function from MACS2. Metaplot and heatmap analysis of ChIP-seq read density at regions of interest was performed with computeMatrix and plotProfile/plotHeatmap from deepTools.
