# Supplementary figures and images for: Distinct roles for CKM–Mediator in controlling Polycomb-dependent chromosomal interactions and priming genes for induction
Source: Nat Struct Mol Biol. 2022 Oct 11;29(10):1000–10. doi: 10.1038/s41594-022-00840-5 (PMC9568430; doi:10.1038/s41594-022-00840-5)

Figure 1A

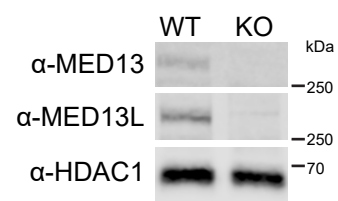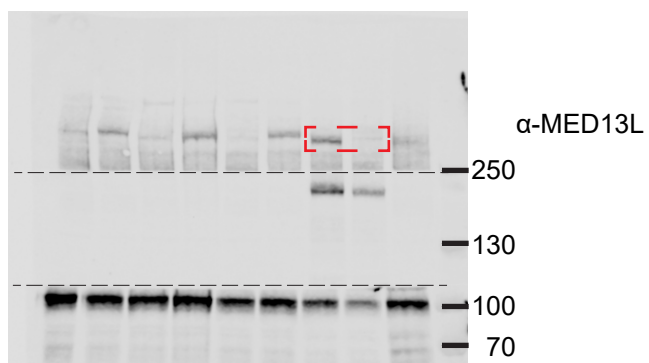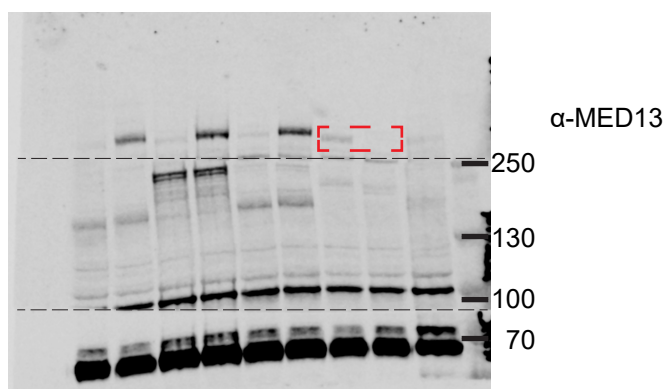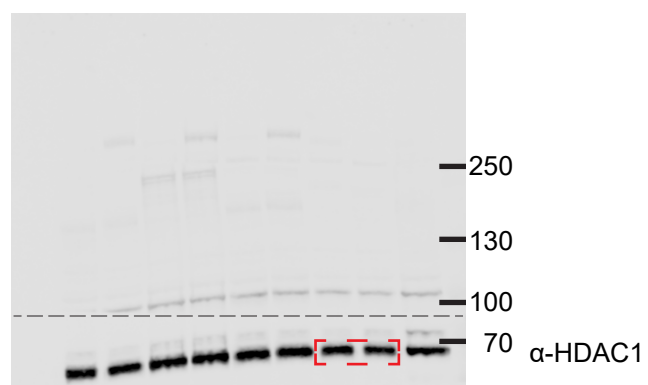

Supplement: Source Data Fig. 1 — Unprocessed Western blots [file 41594_2022_840_MOESM4_ESM.pdf]

# Extended data 1A

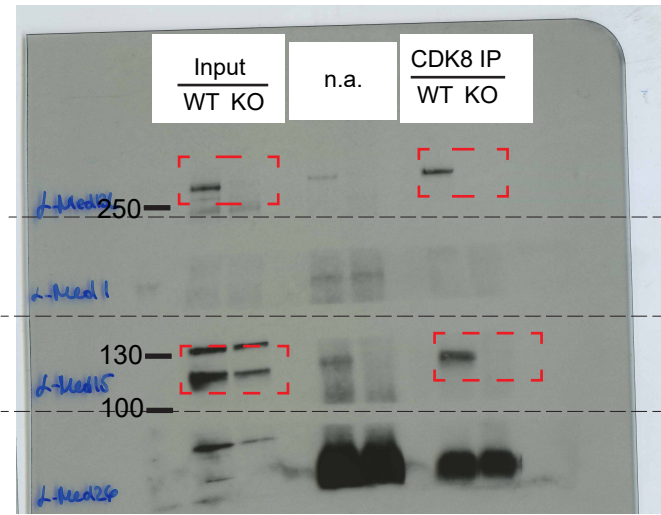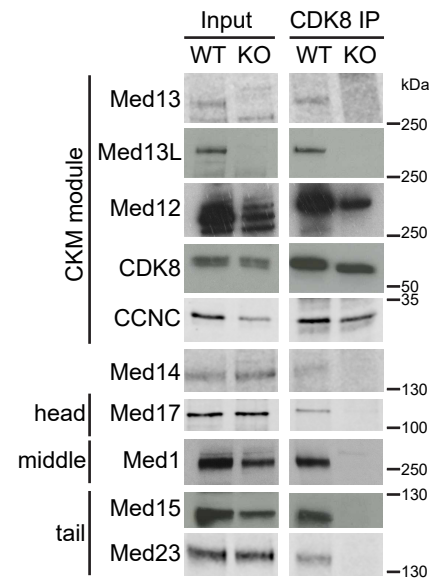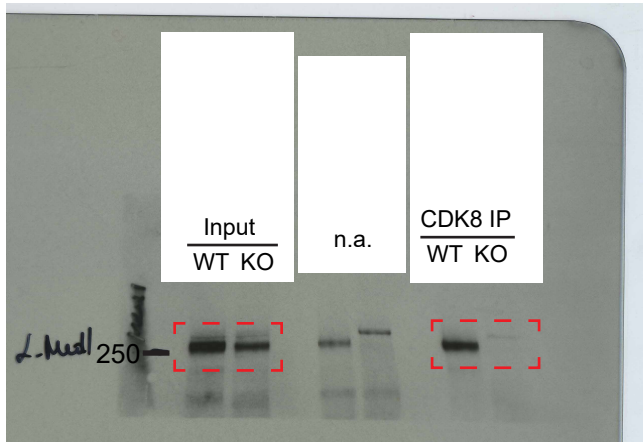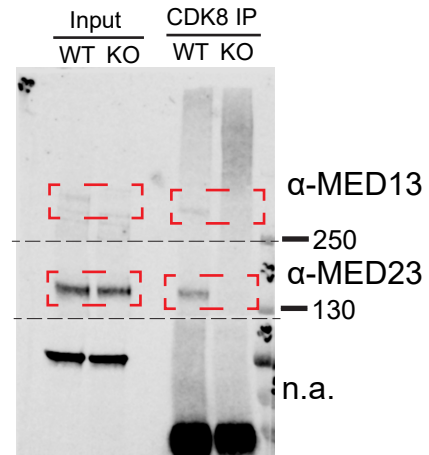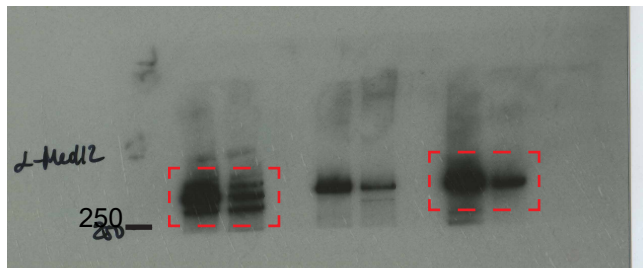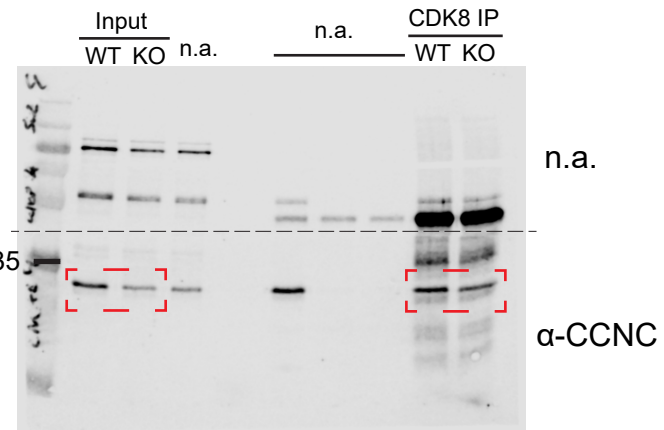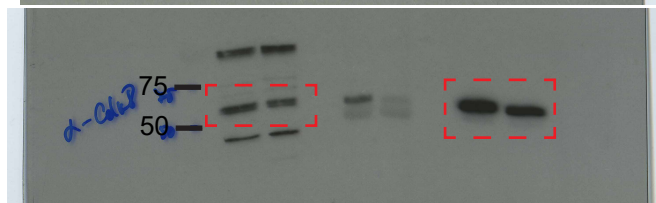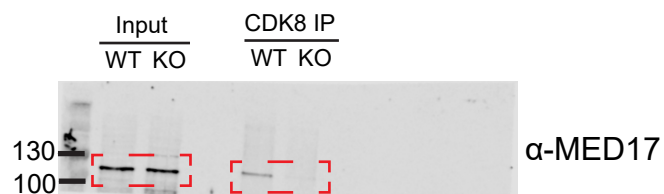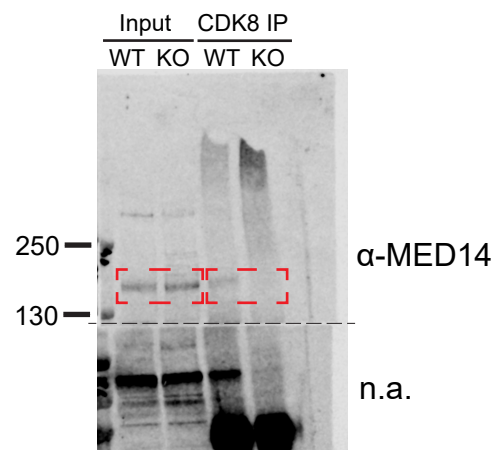

Supplement: Source Data Extended Data Fig. 1 — Unprocessed Western blots [file 41594_2022_840_MOESM5_ESM.pdf]

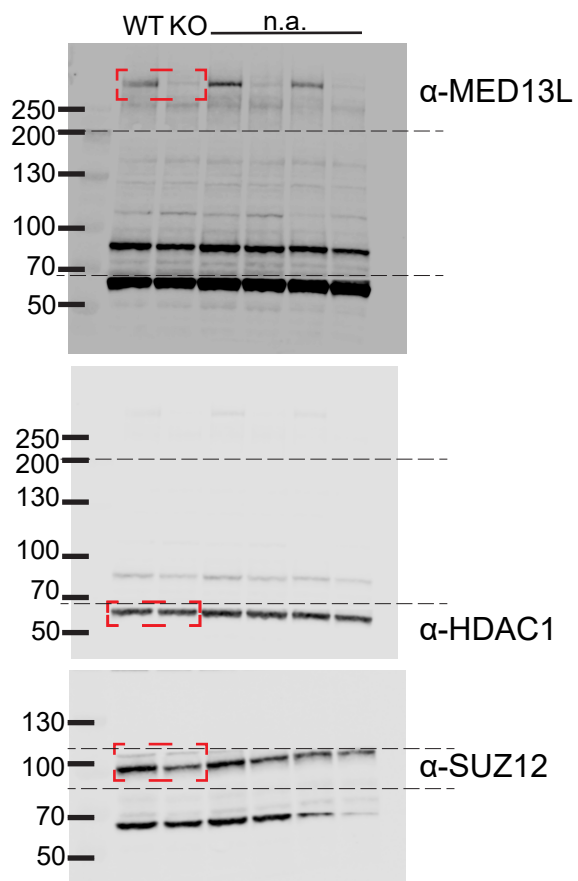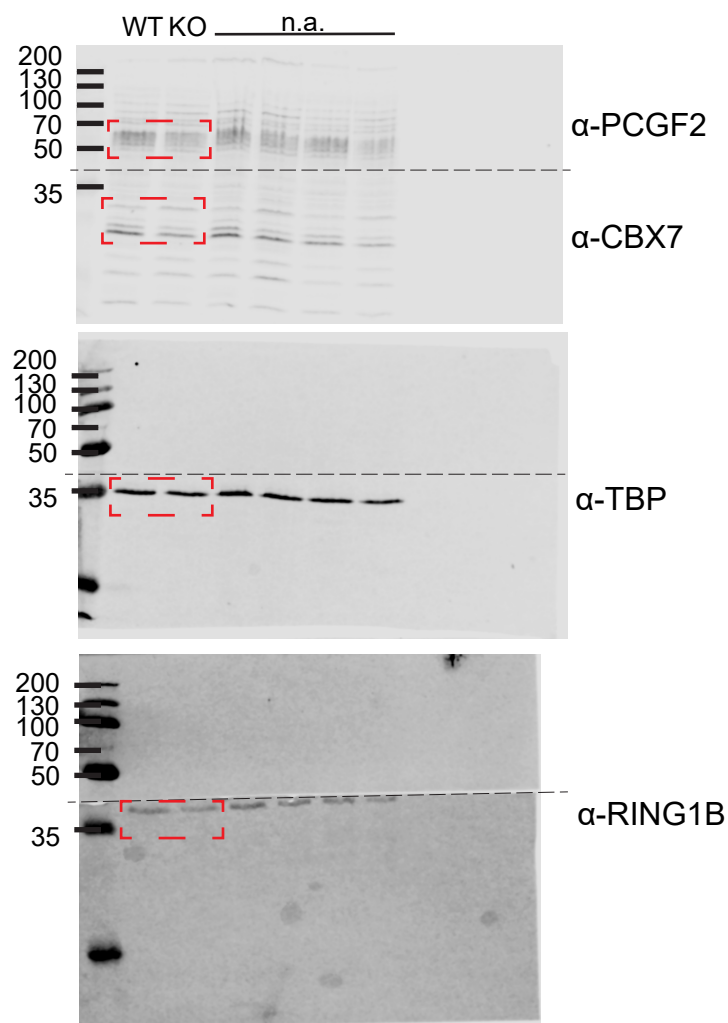

## Extended data 2D

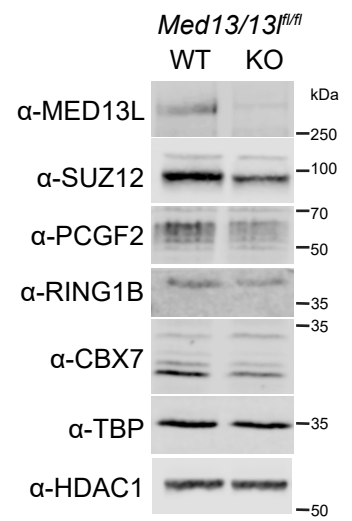

Supplement: Source Data Extended Data Fig. 2 — Unprocessed Western blots [file 41594_2022_840_MOESM6_ESM.pdf]

## Extended data 3A

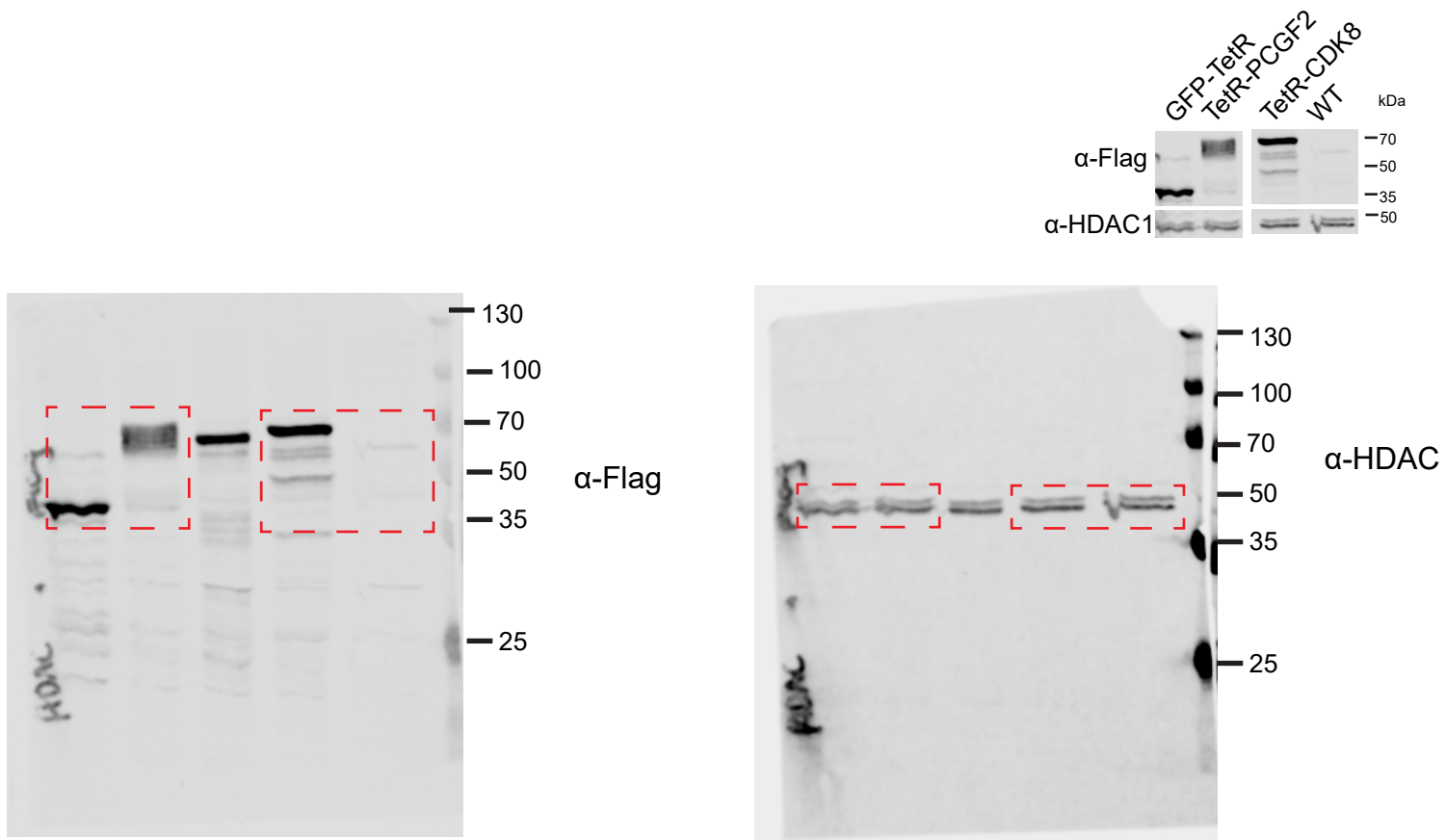

## Extended data 3F

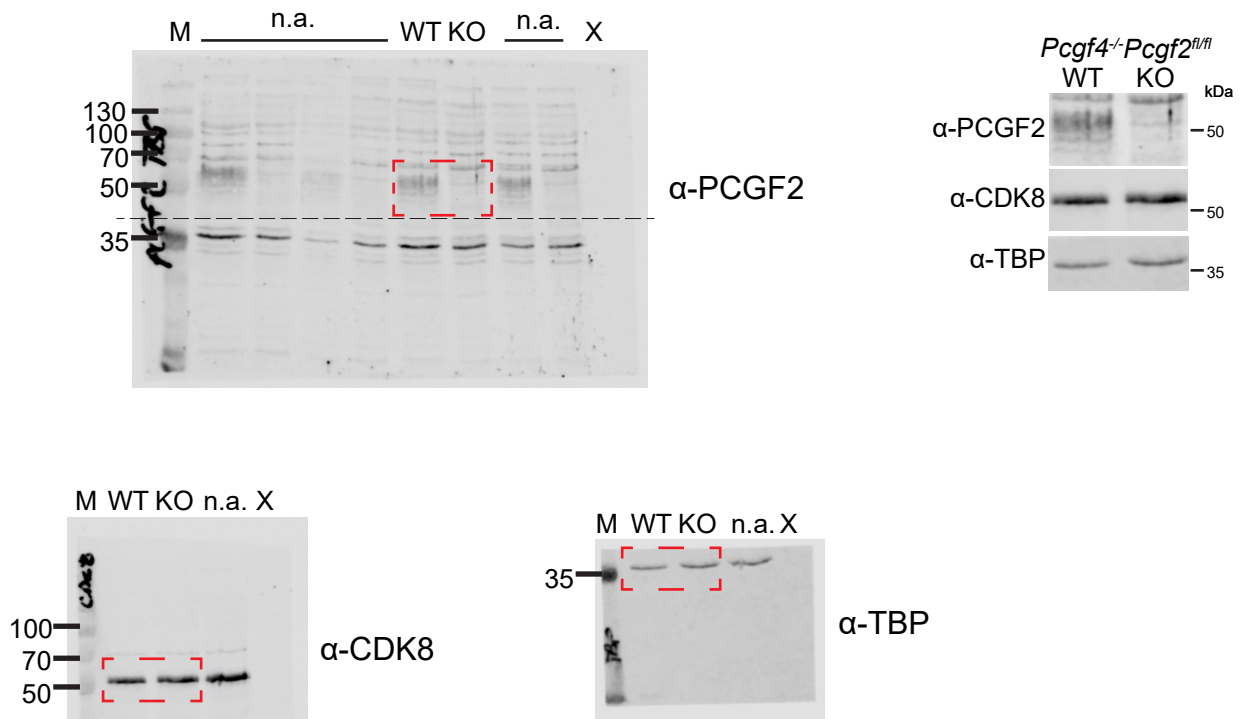

Supplement: Source Data Extended Data Fig. 3 — Unprocessed Western blots [file 41594_2022_840_MOESM8_ESM.pdf]

## Extended data 5B

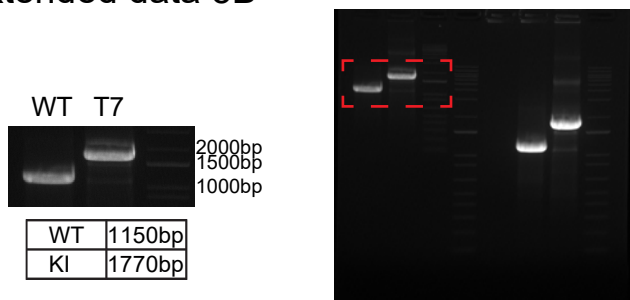

## Extended data 5C

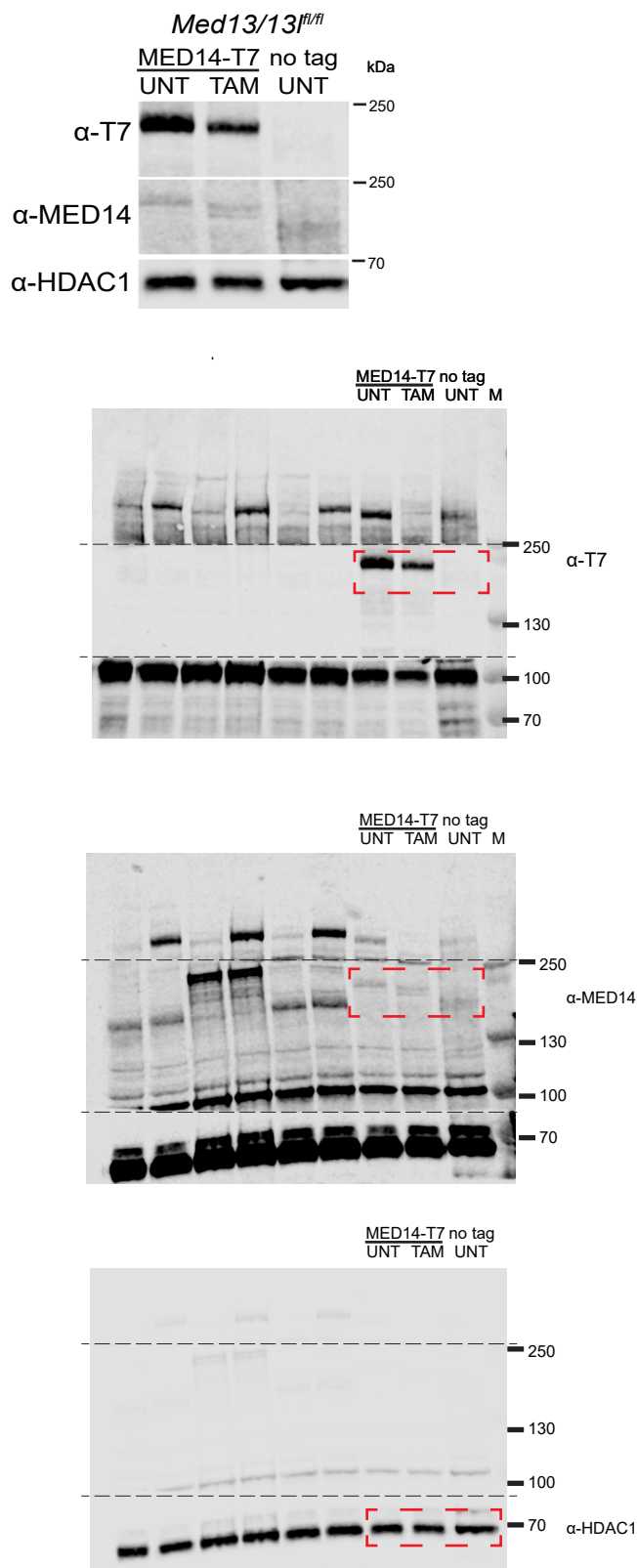

## Extended data 5D

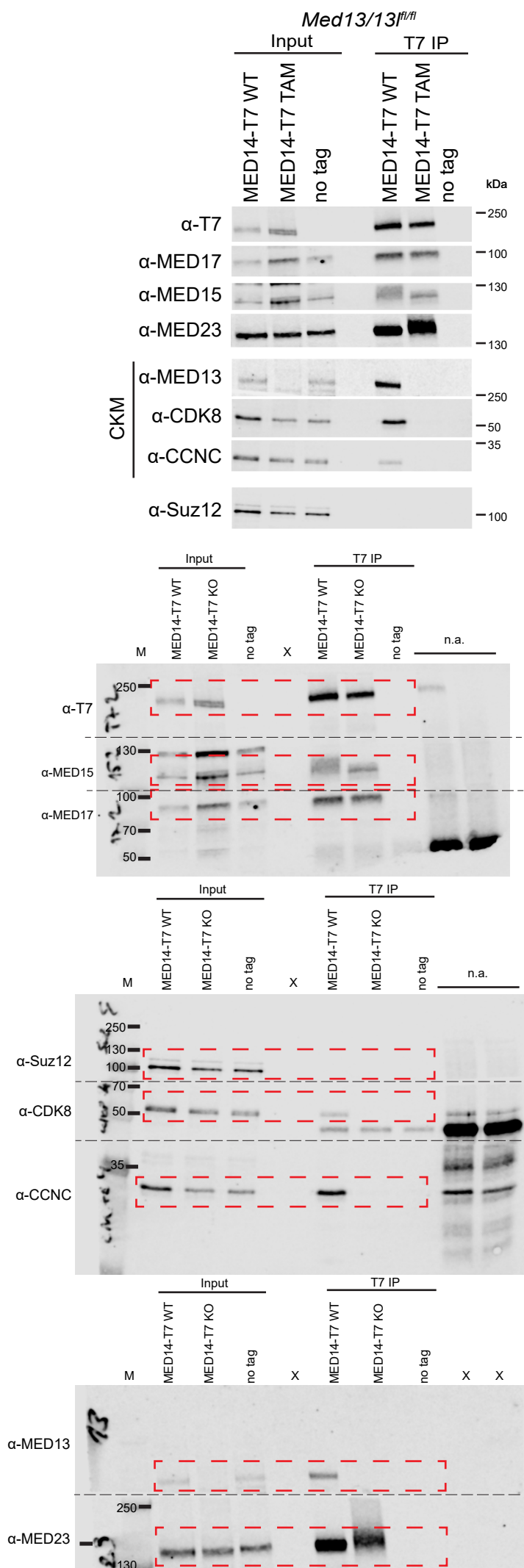

Supplement: Source Data Extended Data Fig. 5 — Unprocessed Western blots and gels [file 41594_2022_840_MOESM10_ESM.pdf]
